# A comparison of course-related stressors in undergraduate problem-based learning (PBL) versus non-PBL medical programmes

**DOI:** 10.1186/1472-6920-9-60

**Published:** 2009-09-13

**Authors:** Alexander D Lewis, Darryl A Braganza Menezes, Helen E McDermott, Louise J Hibbert, Sarah-Louise Brennan, Elizabeth E Ross, Lisa A Jones

**Affiliations:** 1 Medical School, College of Medical and Dental Sciences, University of Birmingham, Birmingham, UK

## Abstract

**Background:**

Medical students report high levels of stress related to their medical training as well as to other personal and financial factors. The aim of this study is to investigate whether there are differences in course-related stressors reported by medical students on undergraduate problem-based learning (PBL) and non-PBL programmes in the UK.

**Method:**

A cross-sectional study of second-year medical students in two UK medical schools (one PBL and one non-PBL programme) was conducted. A 16-question self-report questionnaire, derived from the Perceived Medical Student Stress Scale and the Higher Education Stress Inventory, was used to measure course-related stressors. Following univariate analysis of each stressor between groups, multivariate logistic regression was used to determine which stressors were the best predictors of each course type, while controlling for socio-demographic differences between the groups.

**Results:**

A total of 280 students responded. Compared to the non-PBL students (N = 197), the PBL students (N = 83) were significantly more likely to agree that: they did not know what the faculty expected of them (Odds Ratio (OR) = 0.38, p = 0.03); there were too many small group sessions facilitated only by students resulting in an unclear curriculum (OR = 0.04, p < 0.0001); and that there was a lack of opportunity to explore academic subjects of interest (OR = 0.40, p = 0.02). They were significantly more likely to disagree that: there was a lack of encouragement from teachers (OR = 3.11, p = 0.02); and that the medical course fostered a sense of anonymity and feelings of isolation amongst students (OR = 3.42, p = 0.008).

**Conclusion:**

There are significant differences in the perceived course-related stressors affecting medical students on PBL and non-PBL programmes. Course designers and student support services should therefore tailor their work to minimise, or help students cope with, the specific stressors on each course type to ensure optimum learning and wellbeing among our future doctors.

## Background

Since the publication of 'Tomorrow's Doctors'[1], there has been a progressive shift in the way medical education is delivered, moving from traditional, didactic lecture-based teaching methods towards a more 'problem-based' approach. In problem-based learning (PBL), students use 'triggers' from clinical scenarios to define their own learning objectives and inform independent research, the findings of which are refined in group discussions[2]. Knowledge is thus acquired in an active and self-directed way, unconstrained by subject divisions[3]. PBL is being increasingly favoured by medical educationalists, as it has been shown by some to better prepare students for the teamwork, communication skills and patient interaction required in clinical practice[4,5]. However, other studies have concluded that there is no convincing evidence that PBL improves knowledge base and clinical performance[5,6].

Several research papers have shown higher levels of stress among medical students[7] and qualified doctors[8] compared to the general population. This may be detrimental, as long-lasting increased stress can lead to decreased work performance[9], substance abuse[10] and mental health problems[11]. Sources of medical student stress are often grouped into three main areas: academic pressures, social/personal issues, and financial problems[12]. However, a study of stress in first-year medical undergraduates on a PBL curriculum in Glasgow found that the principal stressors were related to medical training (in particular, uncertainty about study behaviour, progress and aptitude) rather than personal problems[13].

Understanding the current stressors faced by medical students, particularly those related to modern medical educational techniques, is a crucial first-step in being able to minimise unnecessary stress when designing medical degree programmes. It will also inform discussion about how stress in medical students is best managed. The aim of this study is to build on previous work by comparing the frequency of a number of course-related stressors reported by contemporary second-year medical students on a PBL and a non-PBL five-year undergraduate medical course.

## Methods

We conducted a cross-sectional, self-report questionnaire study in two UK medical schools in the academic year 2007-2008. Ethical approval was granted prior to study commencement by the University of Birmingham Medical School Education Unit.

All UK medical schools that run a five-year undergraduate medical degree programme were eligible for inclusion, subject to the following exclusion criteria: *i) *medical schools in Scotland as Scottish students do not pay top-up tuition fees, which could be a confounder given that financial burden is an established stressor in students[12,13]; *ii) *London medical schools, due to the higher cost of living in London compared to the rest of the country; *iii) *medical schools with three-year pre-clinical courses, as most run a separate degree in this time which may be associated with different stressors among students.

Sixteen eligible medical schools (N = 16) were invited to participate by email. Non-responders were reminded by telephone at 2 and 4 weeks. Four (25%) refused and five (31%) failed to respond. The seven that agreed to take part (44%) were categorised into two groups based on information about the proportion of core teaching sessions deemed to be problem-based in the second-year academic timetable. This information was provided by the Director of Medical Education or the Dean. Programmes were classified as PBL or non-PBL if overall course content was reported to be ≥50% or <50% PBL respectively. Two were classified as PBL and five as non-PBL. From this list, one medical school from each group (PBL and non-PBL) was selected randomly via a computer. The two participating medical schools remain anonymous as part of the agreed terms for undertaking the study. The overall course content taught via PBL was reported to be 100% in the PBL university where lectures and seminars are used only to support PBL, and 0% in the non-PBL university where there are no PBL sessions.

Second-year students in each medical school were invited to participate. Second-years were chosen to minimise the risk of confounding by non-course-related stressors. In contrast to first-year students, they are less likely to experience stressors related to adapting to a new lifestyle and way of learning[14]. Students in third-year onwards are predominantly hospital-based, and follow a vocational rather than a PBL or non-PBL style of learning. Questionnaires were distributed during second-year plenary sessions in each institution in order to maximise student recruitment and were collected immediately after completion. A detachable Participant Information Sheet was attached to the questionnaire and students were able to ask the researchers any questions about the study prior to deciding whether or not to participate. Questionnaires were completed voluntarily and anonymously.

### Outcome Measures

A 16-question, self-report questionnaire to measure course-related stressors was developed by combining parts of two existing scales: the Perceived Medical Student Stress scale (PMSS)[12] and the Higher Education Stress Inventory (HESI)[15]. Both questionnaires had shortcomings for the purpose of this study, therefore relevant questions from each were selected. The HESI surveyed issues outside the remit of this study (such as concerns that 'a cold and impersonal attitude is enhanced by education' and 'the professional role is in conflict with personal values'). The older PMSS scale contained questions that were less relevant to modern medical courses and also outside the remit of this study (such as concerns that 'medical school is more of a threat than a challenge' and 'students will be unable to endure the long hours and responsibilities associated with clinical training and practice'). Response options were presented on a 4-point Likert scale (strongly agree/agree/disagree/strongly disagree). The questionnaire was piloted on 30 third-year medical students at one of the potential universities to assess acceptability, understanding and average completion time. Subsequently, two questions (4 and 8) were rephrased to aid comprehension, without changing the essence of their meaning. The final set of questions is presented in Table 1. Demographic information collected included: age, sex, English as a first language, international student status, previous degrees, housing, physical or mental illness, financial stress, and a measure of recent (previous 6 months) adverse life events (e.g. illness, bereavement, severe relationship problems) based on the Brief Life Event Questionnaire (BLEQ)[16].

**Table 1:** Frequency of responses for each course-related stressor by course type

**Question**	**Course type**	**Agree**	**Disagree**	**χ^2^**	**Odds Ratio**
		**N (%)**	**N (%)**	**(P-Value)**	**(95% CI)**
1. *I am enjoying being at medical school*	PBL	77 (*96.2%*)	3 (*3.8%*)	0.16	0.916
	Non-PBL	188 (*95.9%*)	8 (*4.1%*)	(0.898)	(0.237 - 3.543)
2. *I feel studying this medical course puts me under significant amounts of stress at the moment*	PBL	51 (*63.0%*)	30 (*37.0%*)	1.098	1.339
	Non-PBL	132 (*69.5%*)	58 (*30.5%*)	(0.295)	(0.775 - 2.312)
**3. *I do not know what the faculty expect of me***	**PBL**	**32 (*39.0%*)**	**50 (*61.0%*)**	**11.338**	**0.383**
	**Non-PBL**	**38 (*19.7%*)**	**155 (*80.3%*)**	**(0.001**)	**(0.217 - 0.676)**
**4. *There are too many small group sessions facilitated only by students, which results in an unclear curriculum***	**PBL**	**23 (*28.4%*)**	**58 (*71.6%*)**	**35.449**	**0.096**
	**Non-PBL**	**7 (*3.7%*)**	**184 (*96.3%*)**	**(< 0.0001)**	**(0.039 - 0.235)**
5. *I am concerned that I will be unable to master the entire pool of medical knowledge*	PBL	60 (*75.0%*)	20 (*25.0%*)	1.487	0.694
	Non-PBL	129 (*67.5%*)	62 (*32.5%*)	(0.223)	(0.385 - 1.251)
6. *Medical school is more competitive than I expected*	PBL	34 (*42.0%*)	47 (*58.0%*)	0.692	1.248
	Non-PBL	93 (*47.4%*)	103 (*52.6%*)	(0.406)	(0.740 - 2.105)
7. *I feel the course relies on passive reception of knowledge rather than active learning*	PBL	22 (*27.2%*)	59 (*72.8%*)	1.295	1.395
	Non-PBL	65 (*34.2%*)	125 (*65.8%*)	(0.255)	(0.785 - 2.476)
**8. *I feel that the type of education I'm receiving is not giving me adequate preparation for clinical years***	**PBL**	**15 (*18.3%*)**	**67 (*81.7%*)**	**7.348**	**0.351**
	**Non-PBL**	**14 (*7.3%*)**	**178 (*92.7%*)**	(**0.007**)	**(0.161 - 0.767)**
9. *I feel there is a lack of time to carry out personal study based on course content*	PBL	39 (*47.6%*)	43 (*52.4%*)	1.233	0.745
	Non-PBL	77 (*40.3%*)	114 (*59.7%*)	(0.267)	(0.442 - 1.254)
**10. *I feel there is a lack of opportunity to explore academic subjects of interest***	**PBL**	**41 (*50.0%*)**	**41 (*50.0%*)**	**6.354**	**0.512**
	**Non-PBL**	**66 (*33.8%*)**	**129 (*66.2%*)**	**(0.012**)	**(0.303 - 0.865)**
**11. *I feel there is a lack of encouragement from teachers***	**PBL**	**22 (*26.8%*)**	**60 (*73.2%*)**	**5.262**	**1.934**
	**Non-PBL**	**78 (*41.5%*)**	**110 (*58.5%*)**	**(0.022)**	**(1.096 - 3.413)**
12. *I feel there is a lack of feedback from teachers*	PBL	50 (*61.7%*)	31 (*38.3%*)	0.752	1.27
	Non-PBL	129 (*67.2%*)	63 (*32.8%*)	(0.386)	(0.740 - 2.178)
**13. *The medical course fosters a sense of anonymity and feelings of isolation amongst students***	**PBL**	**16 (*20.3%*)**	**63 (*79.7%*)**	**11.521**	**2.848**
	**Non-PBL**	**81 (*42.0%*)**	**112 (*58.0%*)**	**(0.001**)	**(1.534 - 5.287)**
**14. *Medical training controls my life and leaves little time for other activities***	**PBL**	**36 (*43.9%*)**	**46 (*56.1%*)**	**4.92**	**0.548**
	**Non-PBL**	**57 (*30.0%*)**	**133 (*70.0%*)**	**(0.027)**	**(0.321 - 0.935)**
15. *I feel there is a lack of support from peers*	PBL	14 (*17.3%*)	67 (*82.7%*)	2.617	0.547
	Non-PBL	20 (*10.3%*)	175 (*89.7%*)	(0.106)	(0.261 - 1.145)
**16. *This course conforms to my prior expectations***	**PBL**	**52 (*65.0%*)**	**28 (*35.0%*)**	**6.044**	**2.046**
	**Non-PBL**	**152 (*79.2%*)**	**40 (*20.8%*)**	**(0.014**)	**(1.150 - 3.642)**

### Data Analysis

Data were analysed using SPSS (Version 16.0). Non-parametric analyses were conducted due to significant deviations from normality in the data. Demographics were compared between the groups using Pearson's chi-squared tests and Mann-Whitney U tests. Each perceived stressor was compared between groups using Pearson's chi-squared test and crude odds ratios were calculated. To ensure that there were adequate numbers in each cell for meaningful statistical analysis, the 4-point Likert scales were dichotomised into two broad categories for each of the 16 potential course-related stressors. These were 'agree' (comprising strongly agree/agree) and 'disagree' (comprising strongly disagree/disagree). Relationships between socio-demographic variables and each potential stressor were examined in both groups separately using chi-squared and Mann-Whitney U tests, depending on data type. Binary logistic regression using backward stepwise likelihood-ratio for variable selection was carried out to determine the best predictors of group membership (PBL v non-PBL). All socio-demographic variables and course-related stressors that were significantly different between groups in univariate analyses were entered into the model.

## Results

### Response rates

At the PBL university 86 out of 157 students (55%) were present at the plenary session. Of these, 83 (97%) responded. At the non-PBL university 198 out of 231 students (86%) were present at the lecture. Of these, 197 (99%) responded. There was no statistically significant difference between the proportion of males and females in our sample and in the entire year group at either university (χ^2 ^= 0.088, p = 0.766 PBL university; χ^2 ^= 0.001, p = 0.979 non-PBL university).

### Demographics

Socio-demographic variables were compared between the two groups (Table 2). Compared to students at the non-PBL university, those at the PBL university were significantly older (p < 0.0001), and were more likely to have a previous degree (p < 0.0001), live in a family home (p < 0.0001) and have a recent adverse life event (p = 0.03). Significantly fewer students at the PBL university reported English as their first language, compared to the non-PBL university (p = 0.001). There were no significant differences between groups on other socio-demographic variables.

**Table 2:** Socio-demographic comparison between students on the PBL and non-PBL programmes

**Demographic**		**PBL**	**Non-PBL**	**Test Value**	**P-Value**
		**N = 83**	**N = 197**	**(Chi-Squared if not otherwise specified)**	
**Age**	**Median**	**22**	**20**	**Kolmogorov Smirnov Z 2.545**	**< 0.0001**
	**IQR^¤^**	**6**	**1**	**Mann Whitney U 5108.5**	**< 0.0001**
	**Range**	**32 (19 - 51)**	**22 (19 - 41)**		
Sex	Male	28 (*33.7%*)	77 (*39.1%*)	0.714	0.398
	Female	55 (*66.3%*)	120 (*60.9%*)		
**Previous degree**	**Yes**	**40 (*48.2%*)**	**33 (*16.8%*)**	**29.951**	**< 0.0001**
	**No**	**43 (*51.8%*)**	**164 (*83.2%*)**		
International student	Yes	12 (*14.5%*)	18 (*9.2%*)	1.69	0.194
	No	71 (*85.5%*)	178 (*90.8%*)		
**English as first language**	**Yes**	**70 (*84.3%*)**	**188 (*95.9%*)**	**11.236**	**0.001**
	**No**	**13 (*15.7%*)**	**8 (*4.1%*)**		
**Accommodation**	**UA^¤^**	**4 (*4.8%*)**	**16 (*8.1%*)**	**22.446**	**< 0.0001**
	**PRA^¤^**	**59 (*71.1%*)**	**171 (*86.8%*)**		
	**FH^¤^**	**7 (*8.4%*)**	**3 (*1.5%*)**		
	**Other**	**13 (*15.7%*)**	**7 (*3.6%*)**		
Physical illness	Yes	7 (*8.4%*)	22 (*11.2%*)	0.47	0.493
	No	76 (*89.6%*)	175 (*88.8%*)		
Mental illness	Never	74 (*89.2%*)	183 (*92.9%*)	1.097	0.578
	Previous	3 (*3.6%*)	5 (*2.5%*)		
	Current	6 (*7.2%*)	9 (*4.6%*)		
**Recent life events**	**Yes**	**22 (*26.8%*)**	**31 (*15.7%*)**	**4.63**	**0.031**
	**No**	**60 (*73.2%*)**	**166 (*84.3%*)**		
Financial stress	Yes	19 (*22.9%*)	38 (*19.4%*)	0.44	0.507
	No	64 (*77.1%*)	158 (*80.6%*)		

^¤ ^Abbreviations: IQR = Interquartile Range; UA = University Accommodation; PRA = Private Rented Accommodation; FH = Family Home

### Perceived stressors

We compared the frequency of responses for each of the 16 perceived stressors between students at the two universities (Table 1). The groups responded significantly differently to 8 out of 16 questions (50%). Compared to students at the non-PBL university, significantly more students at the PBL university agreed that: *i) *they did not know what the faculty expected of them (Q3) (p = 0.001); *ii) *there were too many small group sessions facilitated only by students, resulting in an unclear curriculum (Q4) (p < 0.0001); *iii) *the education they received was not giving them adequate preparation for clinical years (Q8) (p = 0.007); *iv) *medical training controls their life, leaving little time for other activities (Q14) (p = 0.03); and, *v) *there was a lack of opportunity to explore academic subjects of interest (Q10) (p = 0.01). Significantly fewer students on the PBL course agreed that: *i) *there was a lack of encouragement from teachers (Q11) (p = 0.02); *ii) *the medical course fostered a sense of anonymity and feelings of isolation amongst students (Q13) (p = 0.001); and, *iii) *the course conformed to their prior expectations (Q16) (p = 0.01).

There was no significant difference between the proportion of students on the two course types reporting the three most frequently reported stressors: *i) *70% of all students (75% PBL and 68% non-PBL) were concerned that they would be unable to master the entire pool of medical knowledge (Q5); *ii) *68% (63% PBL and 70% non-PBL) felt that the course was currently putting them under significant amounts of stress (Q2); and, *iii) *66% (62% PBL and 67% non-PBL) felt there was a lack of feedback from teachers (Q12). Although the majority of students in each university disagreed that there was a lack of time to carry out personal study (Q9) and that medical school was more competitive than they expected (Q6), these stressors were still frequently reported (42% and 46% of all students respectively). Importantly, the majority of students in each university agreed that they were enjoying being at medical school (Q1) and that there was not a lack of support from peers (Q15). These were two of the least frequently reported stressors (4% and 12% of all students respectively).

No significant relationships were found between any of the socio-demographic variables and any of the stressors in either group.

### Logistic regression

All socio-demographic variables (age, previous degree, English as a first language, accommodation, and recent life events) and course-related stressors (Q3, Q4, Q8, Q10, Q11, Q13, Q14, and Q16) that were significantly different between groups in univariate analyses were entered into the model. After controlling for socio-demographic differences between the groups, five course-related stressors were found to be significant predictors of PBL versus non-PBL status. The best regression model accounted for 49% of variance (Nagelkerke R^2 ^= 0.486) and correctly classified 83% of participants as PBL or non-PBL. The significant predictors of PBL status were: *i) *more likely to agree that they did not know what the faculty expected of them (Q3) (OR = 0.38, 95% CI = 0.17-0.89, p = 0.03); *ii) *more likely to agree that there were too many small group sessions facilitated only by students, resulting in an unclear curriculum (Q4) (OR = 0.04, 95% CI = 0.008-0.16, p < 0.0001); *iii) *more likely to agree that there was a lack of opportunity to explore academic subjects of interest (Q10) (OR = 0.40, 95% CI = 0.18-0.87, p = 0.02); *iv) *less likely to agree that there was a lack of encouragement from teachers (Q11) (OR = 3.11, 95% CI = 1.24-7.81, p = 0.02); and, *v) *less likely to agree that the medical course fostered a sense of anonymity and feelings of isolation amongst students (Q13) (OR = 3.42, 95% CI = 1.38-8.47, p = 0.008).

## Discussion

This is the first comparison of course-related stressors between medical students on PBL and non-PBL programmes in the UK. Our questionnaire was based on two existing measures of course-related stress in medical students, and was piloted to ensure adequate comprehension and acceptability. The response rate of students present at the plenary teaching session in both universities was high and our analysis suggests that both groups were representative of the study population in terms of gender. Although our two groups were significantly different on a number of socio-demographic variables, we did not find any relationships between these variables and the reporting of stressors and a multivariate analysis was conducted to control for these differences.

### Stressors in PBL compared to non-PBL students

Compared to students on the traditional (non-PBL) programme, significantly more students on the PBL programme felt that they did not know what the faculty expected of them and that there were too many student-facilitated sessions, resulting in an unclear curriculum. Although the majority of PBL students disagreed with these statements, they are clearly potential stressors for a significant minority (39% and 29% PBL students respectively). This could be because of a lack of clearly defined limits related to the learning outcomes of PBL scenarios, resulting in uncertainty as to the depth of learning required. This could lead to student insecurity and lack of confidence about whether they have learnt the relevant concepts in inadequate, or even excessive, detail for assessments, adding to the established fear among medical students of making a mistake[12]. The feeling of insufficient completion of work may lead to a greater amount of time dedicated to studying, possibly explaining why they were more likely to feel that there was a lack of opportunity to explore academic subjects of interest (50% PBL students agreed with this statement compared with 34% non-PBL students). Our findings concur with those of Moffat et al, which showed that PBL medical students in Glasgow felt uncertain about their study behaviour, progress and aptitude[13].

Our study also revealed that, compared to the non-PBL students, significantly fewer students on the PBL programme felt that there was a lack of encouragement from teachers (27% PBL students versus 42% non-PBL students) and an increased sense of anonymity and isolation among students (20% PBL students versus 42% non-PBL students). The data we received from Directors of Medical Education/Deans at the universities showed that, as expected, the non-PBL programme has fewer small group teaching sessions than the PBL programme, implying that the non-PBL students spend a greater proportion of time as a large year group (which is also larger than the PBL university's year group: 231 versus 157 respectively). Within this setting there is less opportunity for contact with, and encouragement from, tutors and therefore a potential reduction in the sense of individuality amongst students.

### Overall frequencies of reported stressors

We identified large numbers of students at both universities reporting 'significant amounts of stress' due to studying medicine, which correlates with other research[7,17]. In total 19% students 'strongly agreed' and 49% 'agreed' (68% in total agreed) with this statement. However, the majority of students at both universities reported 'enjoying being at medical school', with 56% strongly agreeing with this statement. Concern about acquiring adequate and entire medical knowledge was the most frequently reported stressor on both course types (20% students 'strongly agreed' and 49% students 'agreed', so 69% in total agreed), and has been demonstrated in other research[12]. Another commonly reported stressor among students was the lack of feedback from teachers, as reported above; a finding reinforced by Duffield et al[18]. Despite being one of the more frequently reported stressors, the majority of students (54%) disagreed with the statement that medical school was more competitive than they had expected. This could be due to strong peer relationships, as only a minority of students reported that there was a lack of peer support (67% 'disagreed' and 21% 'strongly disagreed' so 88% in total disagreed) and it is known that such support reduces the feeling of 'peer competition' and is potentially important for managing stress levels[19].

Despite the fact that PBL is considered advantageous for encouraging active learning and that non-PBL courses are often criticised for relying on passive reception of knowledge, it is interesting and heartening that there was no significant difference between the two courses in terms of whether students thought the course relied on passive reception of knowledge (73% students at the PBL university and 66% students at the non-PBL university disagreed with this statement).

### Implications and Recommendations

In this study we have found that, although most students enjoy being at medical school, various course-related stressors affect them and these are related to the type of programme being followed. Medical schools should be informed of the types and frequency of stressors their students perceive so that measures can be taken to help students adopt appropriate coping mechanisms resulting in decreased psychological morbidity[14] and, potentially, increased academic performance[20]. Student support services may be adapted to deliver a more effective system tailored to their students' needs. For example, it may be that PBL students require coaching on attaining a healthy work-life balance and how to cope with setting their own learning goals. Those responsible for PBL programme design should consider making regular assessments of the quality of PBL scenarios to ensure that the learning outcomes students pick out from cues in the problems are consistent with the intended learning outcomes. Students should be reassured that this happens in order to increase self-confidence in their learning. Students should understand the emphasis on continuous learning to eradicate the perception that the entirety of medical knowledge must be instantly mastered. Non-PBL programmes should consider ways of increasing encouragement given to students and reducing feelings of anonymity among the student body. Increasing the number of small-group teaching sessions would seem an obvious first-step to achieving this. Both course types could benefit from increasing the amount of feedback provided by teachers. Furthermore, the role of the teacher within the context of PBL could be redefined to offer more active support to students. We believe our findings are also important for prospective medical students in choosing the most appropriate programme for their needs. For example, a prospective student who finds it difficult to switch off from work and has a highly perfectionistic approach to their studies may be less suited to a PBL programme.

### Limitations

Our findings must be interpreted in light of a number of limitations. The study was based on only two medical schools. There were several reasons given by medical schools that refused to participate, including: questionnaire fatigue among their students, course restructure, and concerns over high stress levels among their students. This study is therefore limited to the specific implementations of PBL and non-PBL curricula at the two participating schools, and cannot be generalised to all other PBL and non-PBL medical programmes in the UK. Moreover, the two programmes contained extremes of PBL content (0% and 100%) so our study does not cover the continuum of PBL content between these two extremes. Although the response rates were high among those students present at the plenary sessions (97% and 99%), a number of students were absent which may bias the data. It is difficult to determine whether students who were more or less stressed would be less likely to attend or to refuse to answer our questionnaire. In addition, the level of perceived anonymity is diminished in a lecture hall environment. A relatively low proportion of students (55%) were present at the plenary session in the PBL university, which may be because the session attended was early in the morning. Other documented reasons for non-attendance at teaching sessions among medical students include a dislike of lecture-based teaching or perceived quality of the session[21]. Other potential confounders that have not been addressed include differences between the schools other than curriculum format or quality of implementation of the curriculum, recent curriculum changes, ethnicity of students, and proportion of students with relatives in the medical profession. Proximity to examinations is also a confounding factor that could not be controlled. Only selected questions were taken from the PMSS and HESI, raising issues regarding our resulting questionnaire's validity. Changing the wording of two of the questions as a consequence of our pilot study feedback, could also detract from the original questionnaires' validity, despite enhancing comprehensibility. The use of a self-report questionnaire limited the types of stressors that could be reported by students. We recognise that 16 variables were examined separately in the univariate analyses; however, concern about multiple testing was addressed by using a multivariate logistic regression.

## Conclusion

Previous research has shown that medical students experience high levels of stress related to their medical education as well as personal and financial issues, and that this can affect their academic performance and wellbeing. This is the first study to show that there are significant differences in perceived course-related stressors between undergraduate medical students on a PBL and a non-PBL programme in the UK. PBL is becoming increasingly popular in medical education, therefore it is important that its associated stressors are understood so that they can be minimised or dealt with effectively. Further research, with a larger and broader sample of medical schools and students, is warranted in this area. Semi-structured interviews would allow the identification of any additional perceived course-related stressors. Longitudinal studies may be of interest in analysing how perceived stressors change throughout students' time at medical school, especially in the transition from the pre-clinical to the clinical stage, on the two different course types.

## Competing interests

The authors declare that they have no competing interests.

## Authors' contributions

ADL, DBM, HEM, LJH, SLB, EER conceived of the study and conducted the data collection. All authors contributed to the design of the study, analysis and interpretation of data and drafting the manuscript. All authors read and approved the final manuscript.

## Authors' information

ADL, DABM, HEM, LJH, SLB, EER are fifth-year medical students and conducted this research as part of their student selected course component. LAJ is a Senior Lecturer in Psychiatry and supervised the research.

## Pre-publication history

The pre-publication history for this paper can be accessed here:


